# Yugra State University Biological Collection (Khanty-Mansiysk, Russia): general and digitisation overview

**DOI:** 10.3897/BDJ.10.e77669

**Published:** 2022-01-20

**Authors:** Nina Filippova, Galina Ganasevich, Ilya Filippov, Anastasia Meshcheryakova, Elena Lapshina, Dmitry Karpov

**Affiliations:** 1 Yugra State University, Khanty-Mansiysk, Russia Yugra State University Khanty-Mansiysk Russia

**Keywords:** bryophyta, fungi, plantae, specimens, digitisation, Western Siberia, data mobilisation

## Abstract

**Background:**

The history of biological collections and digitisation initiatives in northern West Siberia is relatively new due to recent development of the region. The Center for Biodiversity Data Mobilization was established to promote the initiative, led by the Yugra State University. This organisation itself has a relatively young collection of biological specimens, which was, until recently, in a disintegrated state and only partly mobilised. The Yugra State University Biological Collection (YSU BC) currently includes three subdivisions differring by history and taxonomic groups, but also by details of management and storage conditions: the Fungarium, the Bryological collection and the Herbarium collection of YSU.

The paper describes the general structure of the Yugra State University Biological Collection, its history, storage conditions, management practices, geographical, temporal and taxonomical coverage. The paper is underlined by three datasets of the collections databases published in GBIF, which are described in detail. The databases are managed in Specify 6 and 7 software and accessed through Specify Web Portal and through GBIF.

**New information:**

The Yugra State University Biological Collection made an active reorganisation of physical storage conditions and data management recently, providing the model for other collections in the region. This paper describes the history, general structure, management practices and data management of all three parts of this collection for the first time.

Although one part of the collection (Fungarium YSU) was mobilised earlier, last year, we mobilised data of the Bryological and Vascular plants (Herbarium) collections. The three datasets of the corresponding collections in GBIF were increased by about 6000 georeferenced records during the last year.

## Introduction

The history of biological collections in the northern West Siberia is relatively new due to the recent development of the region, except for a few collections. In Khanty-Mansi Autonomous Okrug, there are three recently established universities with young biological collections. Some collections of biological specimens are stored in regional history museums, paleonthological and biological departments. Many protected areas of different status have their biological collections, representing the biodiversity of these areas. The oldest collection in the region is managed by the Malaya Sosva State Nature Reserve (established since 1932). The integration of information about the natural history collections in Yugra Region was initiatated by a series of publications (Belogay and Skuchas 2015, Belogay and Skuchas 2018). The information on biological collections of the northernmost territory of Yamal-Nenets Autonomous Okrug is still scarce, but some amount of specimens could be managed by the collections of protected areas, field stations or in personal collections of researchers.

The plant and fungi diversity of the region in the pre-digital age was studied for at least two centuries by researchers and organisations from other regions or abroad (e.g. the "Flora of Western Siberia" edition for plant diversity research; and the review of the history of mycological research in the region in Filippova et al. (2020b). At present, the GBIF search of digitised records from this area retrieved several major collections: Moscow University Herbarium, Central Siberian Botanical Garden (Novosibirsk), Tyumen State University, Komarov Botanical Institute and others. The total number of digitised collection specimens mobilised through GBIF for northern West Siberia is about 60 K (filtered on the basis of record - "preservedSpecimen", "fossilSpecimen" and geographical boundaries of the region, including three administrative territories of Khanty-Mansi, Yamal-Nenets and Tyumen Regions) (GBIF.org 2021).

The initiatives on data mobilisation of biological collections in northern West Siberia have been steadily on the rise throughout the past few years and described in the earlier publications (Filippova et al. 2019, Filippova et al. 2020). The Center for Biodiversity Data Mobilization was established to promote biodiversity informatics and support biodiversity data mobilisation amongst scientific and nature protection organisations and citizen scientists. As part of this programme, a Biological Collections Portal was developed using Specify software, which currently hosts seven collections from different organisations with a total number about 15 K specimens. Leading this initiative, the Yugra State University serves to provide the platform on university's server and to promote collection data management and mobilisation.

The main goal of this publication was to describe the integrated biological collections of Yugra State University in a single publication. During the last (2021^st^) year, we made an active reorganisation of physical storage conditions and data management, making the history and future goals of this collection more visual.

The YSU BC currently stores about 15000 specimens of fungi, mosses, hepatics and vascular plants (https://fungariumysu.org). It was initially based on private collections from several researchers working on biodiversity assessment in Yugra State University. The collection has been registered in Index Herbariorum since 2016 with an acronym "YSU". In 2017, YSU BC was registered as a data publisher in GBIF.

The main goal of the YSU BC is to develop a reference library of regional biodiversity, support research in systematics, ecology, phylogeny and applied disciplines and manage associated biodiversity information in northern West Siberia. For that purpose, we are developing conditions for physical storage of samples, facilitating their scientific analysis and applying modern computer technologies for storage, analysis and distribution of the collection data.

We encourage researchers working in the area to access their biological collections in YSU BC. A rule for the visitors of the Mukhrino Field Station of Yugra State University is to share their collected material with the host university.

The database of YSU BC is developed using Specify software. The Specify Web Portal is available for the database search, browsing the maps and images and can be downloaded. The database is shared through GBIF by IPT software using MySQL queries to each individual collection, including images of specimens, with a weekly update interval.

### Fungarium YSU

The fungarium collection was started as part of the larger fungi inventory programme in the region and PhD work on fungal communities of raised bogs by Nina V. Filippova around 2010. It was later supplemented by collections from several mycologists making inventory work in the area, exsicata exchange and expeditions in other regions of Russia. The major part of the collection, between 2010-2014, was made during the study of fungal communities of raised bogs, including larger fungi, microfungi on plant debris by direct observation, lignicolous fungi and myxomycetes in bogged habitats. In 2015, a large number of specimens (about 1500) were obtained from a then-launched plot-based monitoring of larger fungi in different forest types in the vicinity of Khanty-Mansiysk. Collecting events from this time to the present supported plot-based monitoring of larger fungi in forests and bogs with an average number of about 700 specimens per year. In 2021, a large collection of about 600 specimens of large fungi was made during the "XI Workshop on the Study of Macromycetes organised by the Commission for the Study of Macromycetes (Russian Botanical Society)" held in Ussuriysk, Primorskiy Kray. Two exchanges of exsiccata were accessed by the YSU Fungarium, which came from Apatity, Institute of North Industrial Ecology Problems (INEP).

The main purpose of the Fungarium is to promote inventory and systematics research of fungi in the taiga zone of Western Siberia. It also serves for education and can be used in different applied disciplines. Since 2021, the classical methods of research in the collection are supplemented by molecular approach with the establishment of the new molecular laboratory of Yugra State University.

The collection is located in the Shapsha Field Station of Yugra State University (Shapsha village, 30 km E from Khanty-Mansiysk).

### Bryological collection of YSU

The purpose of the Bryological collection of YSU was to make a collection of mosses and liverworts, representing various biogeographical areas and the species diversity of the Khanty-Mansi Autonomous Okrug, which would be accessible by bryologists worldwide. The study of the Khanty-Mansi Autonomous Okrug in terms of bryology is still insufficient. The regional checklist of species is quite complete, but frequency data are scarce (including rare and endangered species) due to the small number of surveyed areas.

The growth of the Bryological collection began when Elena D. Lapshina started working in the Khanty-Mansi Autonomous Okrug. The first specimens were collected in 2003-2004 in the vicinity of Khanty-Mansiysk Town. In 2009, the Mukhrino Field Station of Yugra State University was established on the left bank of the Irtysh River, 25 km southwest of Khanty-Mansiysk. As a part of the inventory programme of this Station, about 600 samples of mosses and liverworts were collected, representing the typical bryoflora of the middle taiga of Western Siberia (Lapshina and Pisarenko 2013).

A significant part of the collection of bryophytes (2141 samples) was made during a series of expeditions organised by the Museum of Nature and Man (Khanty-Mansiysk) at the eastern slope of the Subpolar Urals. In 2013, the region of Neroyka mountain – one of the highest peaks of the Ural Mountains, was inventoried (Konstantinova and Lapshina 2014, Lapshina et al. 2015, Lapshina et al. 2016). The upper reaches of the Puyva River were visited in the field season of 2015 (Konstantinova and Lapshina 2017, Skuchas and Lapshina 2018). In 2019, a large collection of bryophytes (683 specimens) was made in upper and middle reaches of the Khulga River (Lapshina et al. 2020). These collections from Khanty-Mansi region were supplemented in 2017 by about 500 specimens of mosses and liverworts from the eastern slope of the Polar Urals in Yamal-Nenets Autonomous Okrug, Yanganape Carbonate Mountain range. A small collection (168 specimens) was made in 2017 in a unique mire ecosystem within the territory of "Vogulka" protected area. Other well-sampled areas include the Natural Parks "Kondinskie Ozera" and "Numto". To date, specimens from about 20 key areas have been accessed in the collection.

The collection is located in the main campus of Yugra State University in Khanty-Mansiysk (Chekhova, 16, 2nd building, 2nd floor, room 203).

### Herbarium, vascular plants collection of YSU

The Herbarium of Yugra State University originated from the geobotanical surveys made in the northern West Siberia. Although initially it was not intended as a systematic collection of plants of that region, the accumulated specimens have been made into a separate subdivision of the Biological Collection of YSU. The Herbarium represents collections made mainly by Elena D. Lapshina, Ilya V. Filippov during the fieldwork in Khanty-Mansi and Yamal-Nenets areas since the beginning of the 21^st^ century.

The collection was not managed and was stored in the "collected in the field" state till recently. Last year, we started the revision, preparation and digitisation of this collection. From a total of about 1.5 K specimens, only about 200 were prepared in sheets and digitised at the time of publication. The whole collection will be prepared in sheets and digitised by the end of 2022.

The collection is located in the main campus of Yugra State University in Khanty-Mansiysk (Chekhova, 16, 2nd building, 2nd floor, room 203).

## Sampling methods

### Sampling description

**Fungarium YSU**

The common procedure of collecting, describing and preserving specimens in the YSU Fungarium follows the protocols described for larger fungi (Mueller et al. 2004). The collection of specimens is made by direct observation and extraction of fruiting structures from the substrate. The two major approaches were used: observation and collection of fruiting structures of larger fungi by the naked eye and lens observations of substrates followed by extraction of smaller fruiting structures of discomycetes, hyphomycetes, pyrenomycetes etc. No pure culture cultivation was used to date. The ongoing organisation of the molecular laboratory of Yugra State University will bring new steps and storage protocols related to molecular systematic and metagenomics approaches.

The general protocol of specimen preparation follows several steps:

1. Fruiting structures are photographed and growing conditions and described in the field.
2. The specimens are extracted from the substrate or collected with the substrate for smaller specimens and packed in aluminium foil to be processed in the laboratory later on the day of collection.
3. Macro-morphological features are described according to schemes required for specific groups, the descriptions being stored in the database.
4. Fruiting structures are dried after processing in a drying oven under 40°C and stored as dry specimens in ziploc bags and labelled kraft-paper envelopes.
5. The collection data and related pictures of fresh fruiting structures and microphotographs are added to the Specify database.

**Figure 1.**
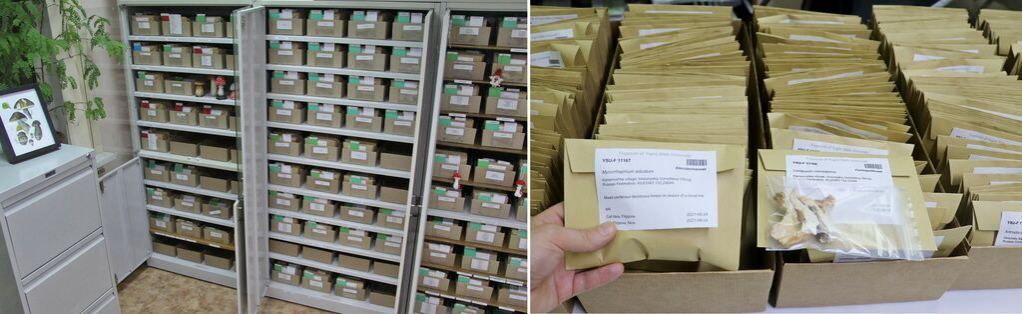
The Fungarium YSU storage conditions and individual envelopes with labels and specimens packed in ziplock bags.

The storage conditions of dried specimens in the Fungarium YSU are shown in the Fig. 1. The vertical filing is applied to arrange paper envelopes on cardboard trays, which have labels and separators to divide different taxa. The cabinets and trays have individual numbers with the corresponding Storage tree in the database to track the exact place of storage. The labels have QR-codes and could be easily retrieved by catalogue number. The collection is organised in systematic (grouping in orders, classes, families, genera and species) and alphabetical (within a taxon) orders. The protocols of the preparation and storage system are visually presented in video presentations on the collection's web site (https://fungariumysu.org/fungarium/storage/).

The collections were made by the specialists or amateur mycologists, the total number of collectors – 50, the majority of specimens in the collection being made by N. Filippova. Identification was performed by a total of 20 specialists through direct work in the Fungarium, in other laboratories with loaned material or making comments on identification through Internet mycological forums. About 25% of the collection specimens are currently not identified to the species rank, including specimens from groups with complex systematics and under-identified field collections.

The digitisation of the Fungarium YSU came online with its development from the beginning. The Fungarium database was managed initially in MS Excel and MS Access and later imported in Specify 6 software. Currently, all specimens of the collection are digitised and mobilised. All specimens were accompanied by *in situ* photographs as an obligatory procedure for the protocol. The images were imported in the database and available for access thorough Specify Web Portal, GBIF (Filippova 2021a) and Mycology Collections Portal. The imaging of dried specimens is not obligatory for fungarium collections, but could be perfomed in the future to provide representation of the storage material.

**Bryological collection YSU**

The major part of bryological specimens was collected during the geobotanical surveys in the area. Well-recognisable species were collected from a few sites within a key area, while cryptic species were collected with more scrutiny. Special attention was paid to river and stream coasts, lake banks, disturbed and rocky habitats, where the specimens were collected without vegetation relevés. This approach provided the most complete list of species being revealed and collected in each key area.

The samples of moss were examined in the field with a hand lens for prior field identification. The samples were extracted and dryed in a herbarium press with the accompanying field label. During the laboratory processing, the samples were transferred in individual paper envelopes with a corresponding label. In case when a sample has several species in admixture, this information was reported in notes.

Identification of most specimens was made in the laboratory using standard microscopical techniques for mosses and liverworts.

**Figure 2.**
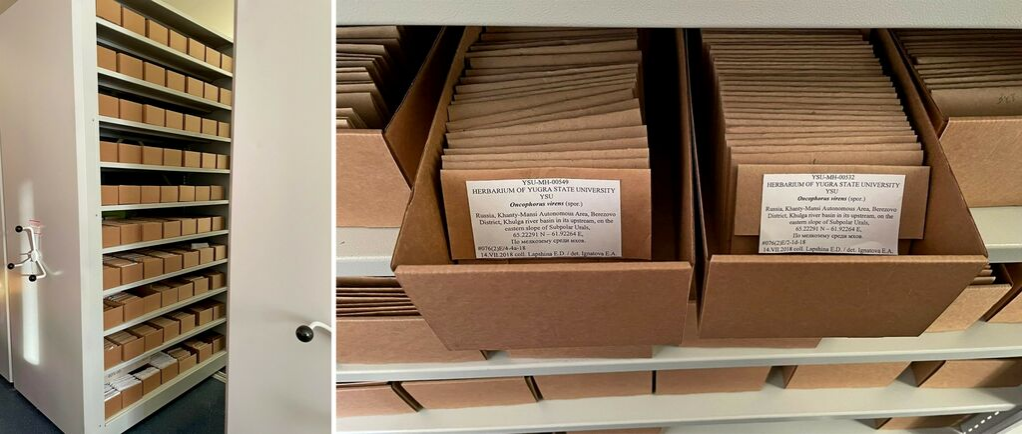
Bryological collection and Herbarium YSU storage conditions (only bryological specimens are shown).

The storage conditions of the specimens in the Brylogical collection YSU are shown in Fig. 2. The specimens in envelopes are arranged in trays in vertical order. The trays are stored in metal filing cabinets with corresponding shelf size.

The digitisation of the Brylogical collection of YSU started during the last couple of years. The label information was manually input to MS Excel and later imported in Specify database. No images of specimens *in situ* or in storage have been made to date. The imaging of dried specimens is not obligatory for bryological collections, as the main identification features remain microscopical. To date, about half of the specimens of the Bryological collection of YSU have been digitised and are accessible through Specify Web Portal, GBIF (Lapshina and Ganasevich 2021) and Moss Flora of Russia (Ivanov et al. 2017).

**Vascular plants collection YSU**

The collection of herbarium specimens was made according to a standard procedure. The plants were collected in a herbarium press and dried under room temperature in newspapers. The montage of herbarium sheets was made later in the laboratory, the standard herbarium technique being followed (Skvortsov 1977).

The herbarium is stored in metal filing cabinets with corresponding shelf size (Fig. 2).

**Figure 3.**
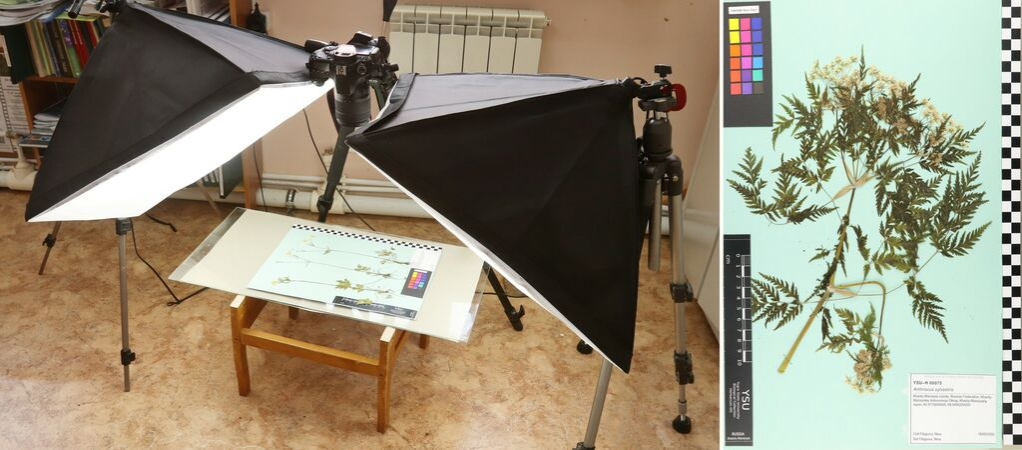
Herbarium YSU imaging protocol, including an imaging system and a resulting image of a herbarium sheet.

The digitisation of this collection started in 2021 from a series of specimens collected during the same year in the vicinity of Khanty-Mansiysk (totally about 130 specimens). We used a hand-made imaging system for small-scale collections digitisation, which included a camera, a tripod and a couple of softboxes. The herbarium sheets were imaged using a Canon 60D camera with the final resolution about 300 dpi, with mounted black/white and colour scales and a barcode (Fig. 3). The digitisation protocol, including images and videos, was published on the web site (https://nwsbios.org/herbarium/).

The Herbarium YSU database with images of herbarium sheets is acccessible through Specify Web Portal and GBIF (Filippova 2021b).

## Geographic coverage

### Description

**Figure 4.**
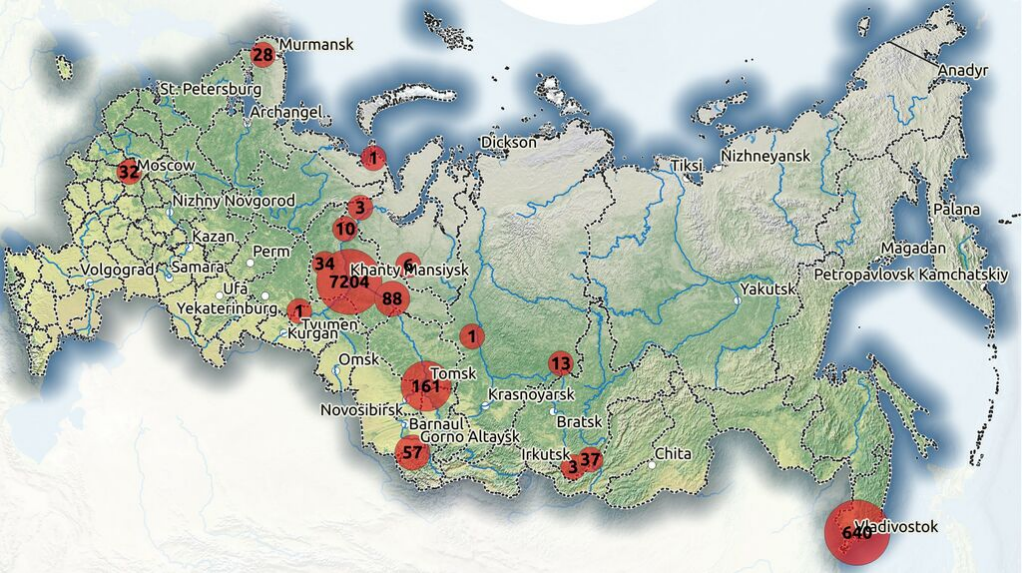
Geographical coverage of the Fungarium collection of Yugra State University.

**Figure 5.**
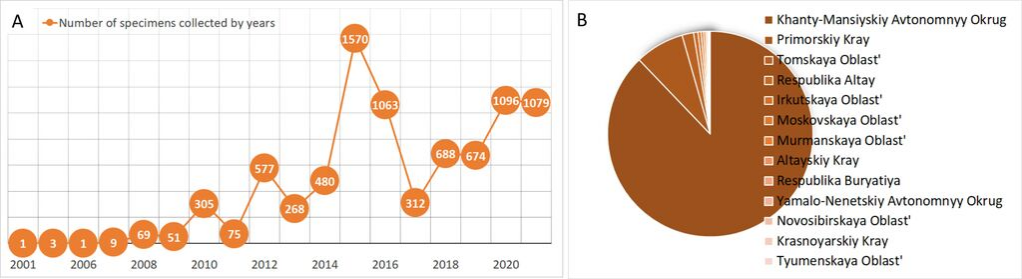
Temporal and geographical coverage of the Fungarium YSU collection: **A** number of specimens collected by years; **B** percentage of specimens collected within administrative borders from a total of 13 regions.

**Specimens deposited in the Fungarium YSU** were collected mainly in the taiga zone of Western Siberia (Fig. 4). The majority of specimens were collected in the adminstrative borders of Khanty-Mansi Autonomous Okrug (87%), 641 specimens from Primorskiy kray and 150 specimens from Tomskaya Oblast'. Several dozen of the specimens were from Irkutskaya Oblast', Moskovskaya Oblast', Murmanskaya Oblast' and from Altay Republic, while the rest of the four regions were represented by a few specimens (Krasnoyarskiy Kray, Novosibirskaya Oblast’, Respublica Buryatia, Tyumenskaya Oblast’) (Fig. 5). Within administrative borders of Yugra Region, six districts were unevenly represented, with the majority (98%) from Khanty-Mansiyskiy District. The two main sampling areas are located in the vicinities of Khanty-Mansiysk, the Shapsha and Mukhrino Field Stations of the Yugra State University (60N 68E). Major vegetation types covered by the collection are coniferous dark taiga forests and their deciduous secondary formations and ombrotrophic raised bogs.

**Bryophyte collection**

**Figure 6.**
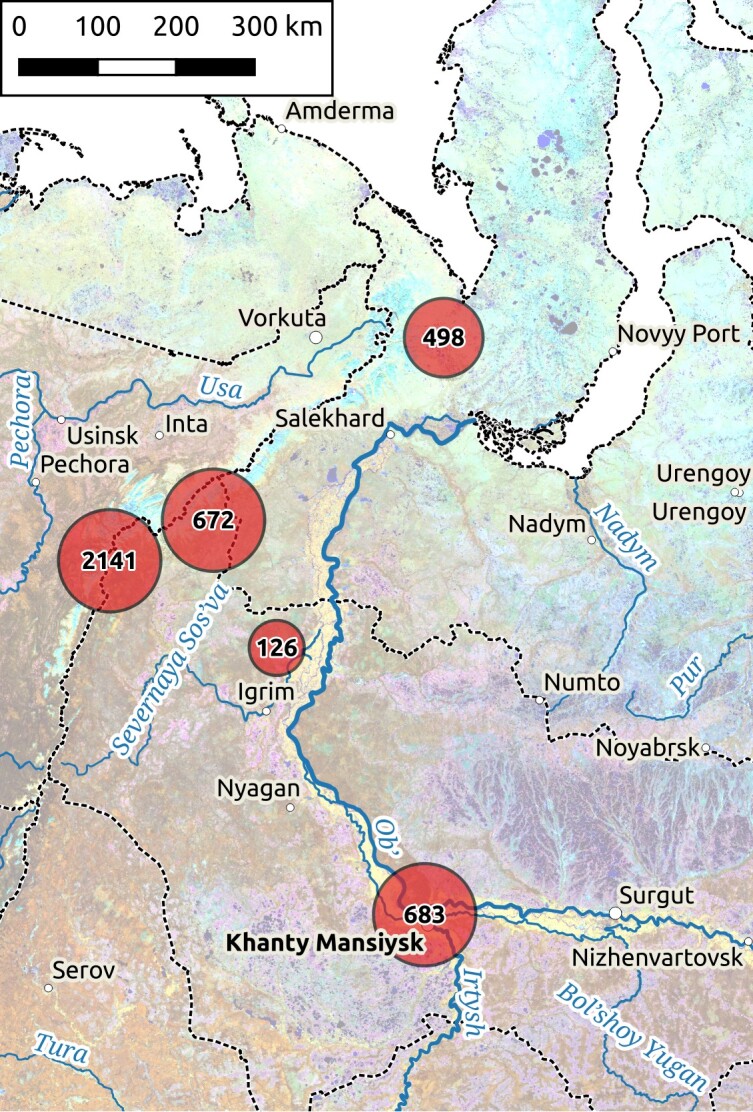
Geographical coverage of the Bryological collection of Yugra State University; only specimens digitised by the time of publication.

**Figure 7.**
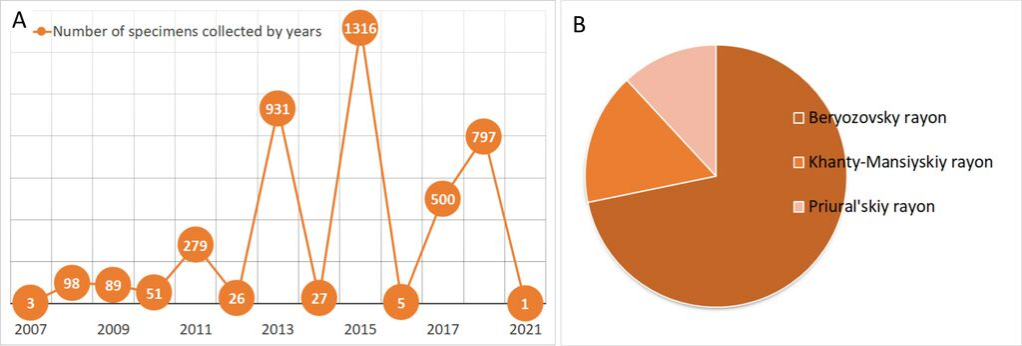
Temporal and geographical coverage of the digitised specimens in Bryological collection YSU: **A** number of specimens collected by years; **B** percentage of specimens collected within the administrative borders from three districts.

**Specimens of the Bryological collection of YSU** were collected mainy in the northern West Siberia (Fig. 6). The majority of digitised specimens were collected from the adminstrative borders of Khanty-Mansi Autonomous Okrug (88%) and 492 specimens from Yamalo-Nenets Autonomous Okrug (Fig. 7). To date, specimens from about 20 key areas have been accessed in the collection, from which about half are currently digitised as shown in the map. The digitised specimens come from eight key areas: the vicinity of the Khanty-Mansiysk town, Mukhrino Field Station of Yugra State University, Neroyka mountain range with upper reaches of the Puyva River, upper and middle reaches of the Khulga River (eastern slope of the Subpolar Urals), Yanganape mountain range (eastern slope of Polar Urals) and the Vogulka protected area.

The geographical coverage of the digitised part of the **Herbarium collection of YSU** is limited to the vicinity of Khanty-Mansiysk Town and Shapsha Village.

### Coordinates

43.09887 and 69.41612 Latitude; 85.783751 and 102.36262 Longitude.

## Taxonomic coverage

### Description

**Figure 8.**
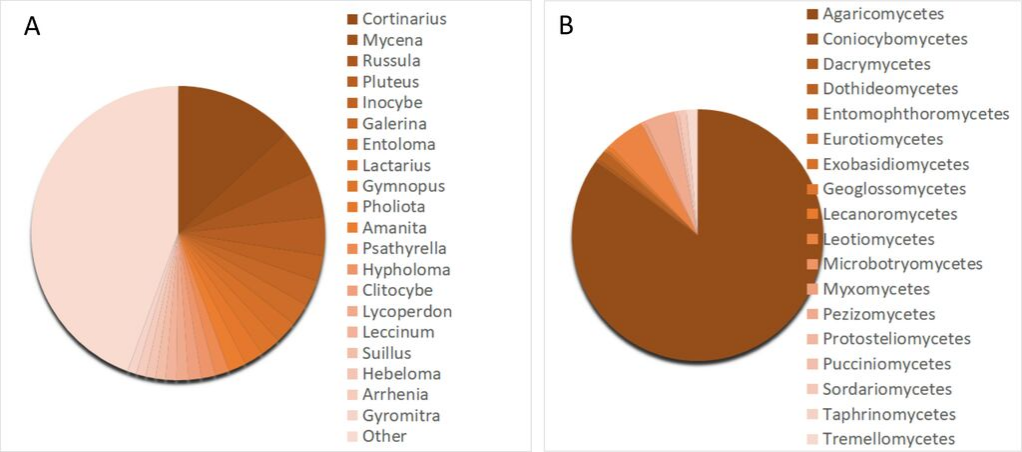
Taxonomic structure of the Fungarium YSU collection: **A** number of specimens per genera; **B** number of specimens per classes.

The taxonomical structure of the **Fungarium YSU** by the time of publication is represented by 1262 species, 520 genera and 181 families (Fig. 8). The majority of specimens in the Fungarium belong to the Basidiomycota (about 86%), less to Ascomycota (12%) with a few specimens from Zygomycota and Myxomycota. The largest classes are the Agaricomycetes (85%), Leotiomycetes (5%) and Pezizomycetes (4%); the other 15 classes are represented by less than 100 specimens. A total of 41 orders are represented, with the majority of specimens from Agaricales (65%), Russulales (8%), Polyporales (4%), Pezizales (4%) and Boletales (4%) and another 55 orders less represented. The specimens of Fungarium YSU belong to 181 families, of which 13% and 7% belong to the families Cortinariaceae and Russulaceae, respectively. They are followed by Mycenaceae (6%), Strophariaceae (6%), Hymenogastraceae (5%), Tricholomataceae (4%), Agaricaceae (4%), Inocybaceae (4%), Pluteaceae (4%), Omphalotaceae (3%) and Entolomataceae (3%), with the rest of the families representing less than 2%. The collection includes 520 genera, notably being *Cortinarius* (1060 specimens), *Mycena* (419 specimens), *Russula* (392), *Pluteus* (342), *Galerina* (227) and *Entoloma* (200), with other genera represented by less than 200 specimens.

**Figure 9.**
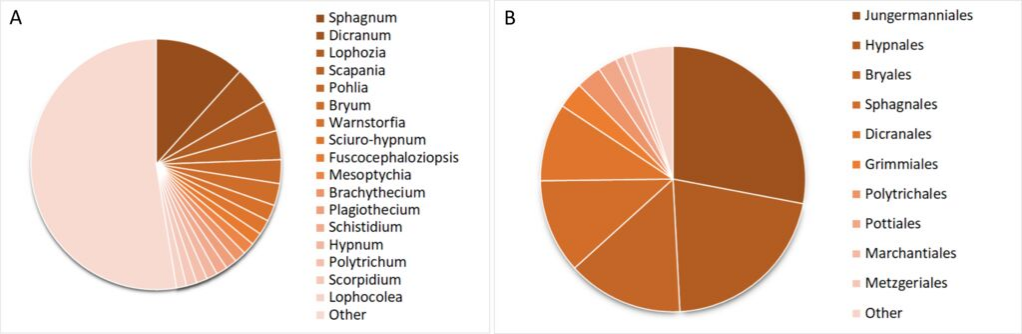
Taxonomic structure of the Bryological collection of YSU: **A** number of specimens per genera; **B** number of specimens per order.

The taxonomical structure of the **Bryological collection of YSU** by the time of publication is represented by 573 species, 195 genera and 77 families (Fig. 9). The majority of specimens belong to Bryophyta (68%), less to Marchantiophyta (32%). Five classes are represented by Bryopsida (57%), Jungermanniopsida (30%), Sphagnopsida (12%), Marchantiopsida and Andreaeopsida (2%, by number of specimens). A total of 27 orders are represented, with the majority of specimens from Jungermanniales (27%), Hypnales (21%), Bryales (14%), Sphagnales (11%) and Dicranales (10%) and another 19 orders less covered. The specimens of Bryological collection of YSU belong to 77 families, with the majority (11%) from Sphagnaceae, followed by Amblystegiaceae (8%), Dicranaceae (8%), Lophoziaceae (6%), Bryaceae (6%), Scapaniaceae (5%), Anastrophyllaceae (5%), Mniaceae (4%), Brachytheciaceae (4%), Grimmiaceae (3%), Polytrichaceae (3%), Hypnaceae (3%) and Cephaloziaceae (3%), with the rest of the families represented by less than 2% of each specimen. The collection includes 209 genera, notably being *Sphagnum* (472 specimens), *Dicranum* (199 specimens), *Lophozia* (163), *Scapania* (152), *Pohlia* (124) and *Bryum* (115), with other genera represented by less than 100 specimens.

## Collection data

### Collection name

Yugra State University Biological Collection

### Collection identifier

YSU-F, YSU-H, YSU-MH

### Parent collection identifier

YSU

### Specimen preservation method

dried

### Curatorial unit

about 15000

## Usage licence

### Usage licence

Creative Commons Public Domain Waiver (CC-Zero)

## Data resources

### Data package title

Yugra State University Biological Collection (YSU BC)

### Resource link

https://www.gbif.org/publisher/fa46e267-4d25-41f5-bbbe-1cd75860b943

### Number of data sets

3

### Data set 1.

#### Data set name

The Fungarium of Yugra State University

#### Number of columns

36

#### Download URL

https://www.gbif.org/dataset/d922b606-6c94-4d51-9277-36c9b03872a7

#### Description

The database of the YSU BC is developed using SPECIFY software. The database structure includes a few dozen related tables and about a hundred used fields. The most important tables include Agents, Collection object, Collection object attribute, Event, Event attribute, Locality, Preparations, DNA sequence, Reference work and others. Three tables with tree structure include Taxonomy, Geography and Storage. The database stores information about collection interactions in corresponding Gift, Loan and Exchange tables. The related attachments (images and other files) to collection object, locality and other items are stored in corresponding Collection object attachments, Collecting event attachments and Locality attachments tables.

To provide data export from the database, we map the Specify fields to DwC archive, which is later accessed by MySQL query of the Integrated Publishing Toolkit, installed on the Yugra State University server. The DwC archive is updated irregularly upon major updates of the database. Each collection of Specify database of the YSU BC is exported and published through GBIF as a separate dataset.

The dataset of the Fungarium YSU is made from 35 mapped fields (see table below). The MySQL query and publication through GBIF is made automatically once a week.

**Data set 1.** 

Column label	Column description
occurrenceID	An identifier for the Occurrence, same as the Catalogue Number.
catalogNumber	A unique identifier of the specimen in collection, the letter prefix (YSU-F) being omitted for simplicity.
otherCatalogNumbers	A list of specimen numbers in other collections, when it was gifted or loaned for study, free text field.
kingdom	The kingdom in which the taxon is classified, from Taxon tree of the database.
class	The class in which the taxon is classified, from Taxon tree of the database.
order	The order in which the taxon is classified, from Taxon tree of the database.
family	The family in which the taxon is classified, from Taxon tree of the database.
genus	The genus in which the taxon is classified, from Taxon tree of the database.
specificEpithet	The specific epithet of the taxon, from Taxon tree of the database.
scientificName	The scientific name of the specimen, linked to the Determination table and Taxon tree in Specify database.
scientificNameAuthorship	The authorship of a scientific name of the specimen, linked to the Determination table and Taxon tree in Specify database.
identifiedBy	A person who determined the specimen, linked to the Determination and Agent tables in Specify database.
dateIdentified	The date of identification, linked to the Determination table in Specify database.
identificationRemarks	Notes on identification, linked to the Determination table in Specify database.
typeStatus	A nomenclatural type of the specimen, a formatted pick list.
continent	The name of the continent, from the Geography tree of the database.
country	The name of the country, from the Geography tree of the database.
stateProvince	The name of the continent, from the Geography tree of the database (in Russian administrative subdivision "okrug", "respublica", "kray" or "oblast'").
county	The name of the county, from the Geography tree of the database (in Russian administrative subdivision "rayon").
decimalLatitude	The geographic latitude.
decimalLongitude	The geographic longitude.
coordinateUncertaintyInMetres	The maximum uncertainty of the geographic coordinate, in metres.
georeferenceSources	A description of the methods used to determine the coordinates (for example: GPS, Google maps etc.).
geodeticDatum	The geodetic datum upon which the geographic coordinates are given.
locality	The specific description of the place of collection.
maximumElevationInmetres	The maximum elevation above sea level, in metres.
eventDate	A date when the collection was made.
locationRemarks	Location description remarks.
habitat	A description of vegetation, relief or other ecological features of the habitat.
occurrenceRemarks	Information about the substrate of collection, free text field.
recordedBy	A list of people responsible for collecting; in Specify database, this field is aggregated from the "Agent" table.
preparations	A list of preparations with their number; in Specify database, this field is aggregated from the "Preparation" table.
associatedMedia	A list of URLs of photographs of the specimen, managed in Specify Attachment Server software.
basisOfRecord	The specific nature of the data record, filled with "preservedSpecimen" value in case of presence of physical specimen or "humanObservation" in case when only photographs presented.
collectionCode	The collection code acronym, YSU-F (internal use, not registered in Index Herbariorum).
institutionCode	The institutional acronyn, YSU (registered in Index Herbariorum).

### Data set 2.

#### Data set name

Bryological collection of Yugra State University

#### Number of columns

36

#### Download URL

https://www.gbif.org/dataset/b73e7171-f1de-47a6-8331-843ed8b541bb

#### Description

The database of the Bryological collection of YSU is developed using Specify software, see detailed description of the database structure under the description of the Fungarium YSU dataset. The data export of 22 fields is made irregularly upon major updates, an auto-publishing through GBIF is made on a weekly basis.

**Data set 2.** 

Column label	Column description
occurrenceID	An identifier for the Occurrence, same as the Catalogue Number.
catalogNumber	A unique identifier of the specimen in collection, the letter prefixes (YSU-F) being omitted for simplicity.
otherCatalogNumbers	A list of specimen numbers in other collections, when it was gifted or loaned for study, free text field.
kingdom	The kingdom in which the taxon is classified, from Taxon tree of the database.
class	The class in which the taxon is classified, from Taxon tree of the database.
order	The order in which the taxon is classified, from Taxon tree of the database.
family	The family in which the taxon is classified, from Taxon tree of the database.
genus	The genus in which the taxon is classified, from Taxon tree of the database.
specificEpithet	The specific epithet of the taxon, from Taxon tree of the database.
scientificName	The scientific name of the specimen, linked to the Determination table and Taxon tree in Specify database.
scientificNameAuthorship	The scientific name authorship.
identifiedBy	A person who determined the specimen, linked to the Determination and Agent tables in Specify database.
dateIdentified	The date of identification, linked to the Determination table in Specify database.
identificationRemarks	Notes on identification, linked to the Determination table in Specify database.
typeStatus	A nomenclatural type of the specimen, pick list formatted.
continent	The name of the continent, from the Geography tree of the database.
country	The name of the country, from the Geography tree of the database.
stateProvince	The name of the continent, from the Geography tree of the database (in Russian administrative subdivision "okrug", "respublica", "kray" or "oblast'").
country	The name of the county, from the Geography tree of the database (in Russian administrative subdivision "rayon").
decimalLatitude	The geographic latitude.
decimalLongitude	The geographic longitude.
coordinateUncertaintyInMetres	The maximum uncertainty of geographic coordinate, in metres.
georeferenceSources	A description of the methods used to determine the coordinates (for example: GPS, Google maps etc.).
geodeticDatum	The geodetic datum upon which the geographic coordinates are given.
locality	The specific description of the place of collection.
maximumElevationInmetres	The maximum elevation above sea level, in metres.
eventDate	A date when the collection was made.
locationRemarks	Additional informaiton about the locality.
habitat	A description of vegetation, relief or other ecological features of the habitat.
occurrenceRemarks	Information about the substrate of collection, free text field.
recordedBy	A list of people responsible for collecting; in Specify database, this field is aggregated from the "Agent" table.
preparations	A list of preparations with their number; in Specify database, this field is aggregated from the "Preparation" table.
associatedMedia	A list of URLs of photographs of the specimen, managed in Specify Attachment Server software installed on the Yugra State University server.
basisOfRecord	The specific nature of the data record, filled with "preservedSpecimen" value in case of presence of physical specimen or "humanObservation" in case when only photographs presented.
collectionCode	The code of the Bryological collection of YSU (YSU-MH) (internal use, not registered in Index Herbariorum).
institutionCode	The institutional acronyn, YSU (registered in Index Herbariorum).

### Data set 3.

#### Data set name

Herbarium of Yugra State University

#### Number of columns

36

#### Download URL

https://www.gbif.org/dataset/0a600588-7e2c-414a-9e33-69d8f35285c2

#### Description

The database of the Herbarium YSU is made using Specify software, see detailed description of the database structure under the description of the Fungarium YSU dataset. The data export of 26 fields is made irregularly upon major updates, an auto-publishing through GBIF is made on a weekly basis.

**Data set 3.** 

Column label	Column description
occurrenceID	An identifier for the Occurrence, same as the Catalogue Number.
catalogNumber	A unique identifier of the specimen in collection, the letter prefixes (YSU-F) being omitted for simplicity.
otherCatalogNumbers	A list of specimen numbers in other collections, when it was gifted or loaned for study, free text field.
kingdom	The kingdom in which the taxon is classified, from Taxon tree of the database.
class	The class in which the taxon is classified, from Taxon tree of the database.
order	The order in which the taxon is classified, from Taxon tree of the database.
family	The family in which the taxon is classified, from Taxon tree of the database.
genus	The genus in which the taxon is classified, from Taxon tree of the database.
specificEpithet	The specific epithet of the taxon, from Taxon tree of the database.
scientificName	The scientific name of the specimen, linked to the Determination table and Taxon tree in Specify database.
scientificNameAuthorship	The scientific name authorship.
identifiedBy	A person who determined the specimen, linked to the Determination and Agent tables in Specify database.
dateIdentified	The date of identification, linked to the Determination table in Specify database.
identificationRemarks	Notes on identification, linked to the Determination table in Specify database.
typeStatus	A nomenclatural type of the specimen, pick list formatted.
continent	The name of the continent, from the Geography tree of the database.
country	The name of the country, from the Geography tree of the database.
stateProvince	The name of the continent, from the Geography tree of the database (in Russian administrative subdivision "okrug", "respublica", "kray" or "oblast'").
country	The name of the county, from the Geography tree of the database (in Russian administrative subdivision "rayon").
decimalLatitude	The geographic latitude.
decimalLongitude	The geographic longitude.
coordinateUncertaintyInMetres	The uncertainy of the geographical coordinate, in metres.
georeferenceSources	A description of the source used to determine the coordinates (for example: GPS, Google maps etc.).
geodeticDatum	The geodetic datum upon which the geographic coordinates are given.
locality	The specific description of the place of collection.
maximumElevationInmetres	The maximum elevation avove sea level, in metres.
eventDate	A date when the collection was made.
locationRemarks	Details about the locality.
habitat	A description of vegetation, relief or other ecological features of the habitat.
occurrenceRemarks	Information about the substrate of collection, free text field.
recordedBy	A list of people responsible for collecting; in Specify database, this field is aggregated from the "Agent" table.
preparations	A list of preparations with their number; in Specify database, this field is aggregated from the "Preparation" table.
associatedMedia	A list of URLs of photographs of the specimen, managed in Specify Attachment Server software installed on Yugra State University server.
basisOfRecord	The specific nature of the data record, filled with "preservedSpecimen" value in case of presence of physical specimen, or "humanObservation" in case when only photographs presented.
collectionCode	The acronym abbreviation of the collection code (YSU-H) (internal use, not registered in Index Herbariorum).
institutionCode	The institutional acronyn, YSU (registered in Index Herbariorum).
